# Wound trauma alters ionizing radiation dose assessment

**DOI:** 10.1186/2045-3701-2-20

**Published:** 2012-06-11

**Authors:** Juliann G Kiang, Bradley R Garrison, True M Burns, Min Zhai, Ian C Dews, Patrick H Ney, Lynnette H Cary, Risaku Fukumoto, Thomas B Elliott, G David Ledney

**Affiliations:** 1 Radiation Combined Injury Program, Armed Forces Radiobiology Research Institute, Bethesda, MD 20889-5603, USA; 2 Department of Radiation Biology, Uniformed Services University of The Health Sciences, Bethesda, MD 20814, USA; 3 Department of Medicine, Uniformed Services University of The Health Sciences, Bethesda, MD 20814, USA

**Keywords:** Radiation, Wound, Combined injury, Lymphocyte, Neutrophil, Platelet, Splenocyte, γ-H2AX, Cytokine, DNA damage, Survivin

## Abstract

**Background:**

Wounding following whole-body γ-irradiation (radiation combined injury, RCI) increases mortality. Wounding-induced increases in radiation mortality are triggered by sustained activation of inducible nitric oxide synthase pathways, persistent alteration of cytokine homeostasis, and increased susceptibility to bacterial infection. Among these factors, cytokines along with other biomarkers have been adopted for biodosimetric evaluation and assessment of radiation dose and injury. Therefore, wounding could complicate biodosimetric assessments.

**Results:**

In this report, such confounding effects were addressed. Mice were given ^60^Co γ-photon radiation followed by skin wounding. Wound trauma exacerbated radiation-induced mortality, body-weight loss, and wound healing. Analyses of DNA damage in bone-marrow cells and peripheral blood mononuclear cells (PBMCs), changes in hematology and cytokine profiles, and fundamental clinical signs were evaluated. Early biomarkers (1 d after RCI) vs. irradiation alone included significant decreases in survivin expression in bone marrow cells, enhanced increases in γ-H2AX formation in Lin^+^ bone marrow cells, enhanced increases in IL-1β, IL-6, IL-8, and G-CSF concentrations in blood, and concomitant decreases in γ-H2AX formation in PBMCs and decreases in numbers of splenocytes, lymphocytes, and neutrophils. Intermediate biomarkers (7 – 10 d after RCI) included continuously decreased γ-H2AX formation in PBMC and enhanced increases in IL-1β, IL-6, IL-8, and G-CSF concentrations in blood. The clinical signs evaluated after RCI were increased water consumption, decreased body weight, and decreased wound healing rate and survival rate. Late clinical signs (30 d after RCI) included poor survival and wound healing.

**Conclusion:**

Results suggest that confounding factors such as wounding alters ionizing radiation dose assessment and agents inhibiting these responses may prove therapeutic for radiation combined injury and reduce related mortality.

## Background

Radiation injury combined with other injuries were observed at Hiroshima and Nagasaki, Japan, where approximately 60% of victims received radiation alone with approximately 40% of victims having other injuries concurrent with radiation injury [1,2]. After the Chernobyl reactor meltdown, 10% of 237 victims exposed to radiation received thermal burns [3]. In animals including mice [4,5], rats [6,7], guinea pigs [8], dogs [9], and swine [10,11], burns and wounds usually increase mortality after otherwise non-lethal irradiation. In mice, irradiation combined with wounds [4,12] decreases body weight, increases the number of bacterial species detected in the tissues, and reduces survival compared to wounds or radiation exposure alone. Consequences of combined injury include acute myelosuppression, immune system inhibition, fluid imbalance, macro/microcirculation failure, massive cellular damage, sepsis, and disruption of vital organ functions, leading to multiple-organ dysfunction syndrome (MODS) and multiple-organ failure (MOF), the most frequent causes of death after combined injury [13-15]. The molecular events underlying combined injury-enhanced mortality include increases in iNOS mRNA and its protein in small intestine and skin and increased cytokine concentrations in serum [4]. These molecular changes suggest potential approaches for the design of countermeasures and therapies as well as possibilities for recovery from combined injury.

Whole-body irradiation induces DNA double strand breaks that lead to ataxia telangiectasia mutated (ATM) phosphorylation. As a result, H2AX is phosphorylated and within seconds becomes γ-H2AX [16,17]. Phosphorylated H2AX is proposed as a biodosimeter for total-body radiation exposure [18]. One day later, whole body irradiation results in lymphocytopenia, neutropenia, and thrombocytopenia [19]. As γ-H2AX, the decrease in numbers of these cells has also been used as a biodosimeter for early assessment of an individual's exposure dose and risk for morbidity and mortality [20]. Increases in IL-6 [4,21] and Bax [17] have also been used as biomarkers for radiation injury [22]. However, Kiang et al. [4,17] reported that wound trauma magnifies radiation-induced cytokine concentrations in blood. Whether wound trauma modified radiation-induced γ-H2AX, lymphocytopenia, neutropenia, and thrombocytopenia was not clear. Like in the case of cytokine increases, we hypothesized that wound trauma enhanced γ-H2AX and hematological responses to radiation. If this hypothesis is supported, then the estimation of radiation dose and risk assessment using these biomarkers will be elusive under the circumstance of the trauma after irradiation.

Herein, we report changes in molecular and cellular biomarkers in mice that were given ^60^Co-γ-irradiation followed by skin-wound trauma, which simulates classical combined injury [23]. Wound trauma enhanced γ-H2AX formation in Lin^+^ bone marrow cells and further reduced γ-H2AX formation in peripheral blood mononuclear cells (PBMCs), numbers of RBCs, neutrophils, and platelets in blood and splenocytes in spleens after irradiation. Increased changes in blood cells and biomarkers were accompanied by clinical signs observed at selected early-to-late time periods. Understanding the combined changes in biomarker concentrations, blood cell numbers, and clinical signs is essential for reconstructing radiation dose and calculating risk assessment after a nuclear accident.

## Results

### Wounding Enhanced Radiation-Induced Mortality, body weight loss, and water intake but slowed wound healing

**Figure 1:**
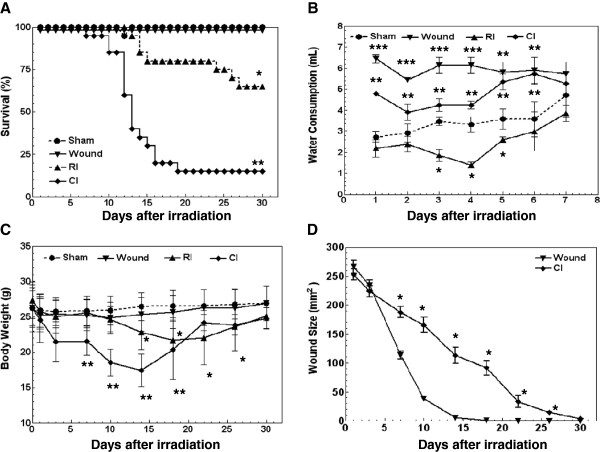
** Wounding enhanced the radiation-induced mortality, water intake, and body weight loss, and delayed wound healing rate.** N = 20 mice per group. (A) Wounding reduced 30-day survival after irradiation. *P < 0.05 vs. sham, wound, and CI groups; **P < 0.05 vs. sham, wound, and RI groups. (B) Wounding and/or radiation increased water consumption during the first 6 d. Radiation alone inhibited water consumption, which returned to normal baseline at 7 d. *P < 0.05 vs. sham, wound, and CI groups; **P < 0.05 vs. sham, RI, and CI groups; ***P < 0.05 vs. sham, RI, and CI groups. (C) Wounding accelerated radiation-induced body weight loss. *P < 0.05 vs. sham, wound, and CI groups; **P < 0.05 vs. sham, wound, and RI groups. (D) Radiation delayed wound healing. *P < 0.05 vs. wound group. RI: Radiation injury at 9.75 Gy; CI: combined injury.

As shown in Figure 1A, death from combined injury and from radiation injury after a dose of 9.75 Gy commenced on day 10 and day 14, respectively. On day 30, mortality was 85% after combined injury and 35% after radiation injury (*P* < 0.05). Wounding alone did not cause any mortality. The data suggest that wounding increased radiation-induced mortality. Radiation dose at 9.75 Gy was LD_85/30_.

In irradiated mice, water intake significantly decreased 1 d later, continued to decrease to 4 d, and returned to normal by 7 d. In both wounded and combined injury mice, water intake increased the first day and persisted for 7 d; wounded mice drank more water than combined injury mice at 2–4 d but not 5–7 d (Figure 1B). The maximal differences in amounts of water occurred at 4 d and were in mL/day/mouse 3.3 ± 0.36, 1.40 ± 0.14, 4.25 ± 0.18, and 6.15 ± 0.38, for sham, irradiated, wounded, and combined injured mice, respectively (*P* < 0.05).

In irradiated mice, body weight was significantly decreased starting day 14 with an approximate rate of −0.27 g/day and reached a nadir at day 22 with approximately 18.5% body-weight reduction. After combined injury, body weight significantly decreased after day 7 with an approximate rate of −0.64 g/day and reached a nadir at day 14 with over 20% of body-weight reduction. Wounding alone did not alter body weight (Figure 1C).

In mice subjected to wounding only, the wound closed within 15 d with an approximate rate of 19 mm^2^/day. When mice were subjected to combined injury, the wound took ≥30 d to close in surviving animals with an approximate rate of 8 mm^2^/day (Figure 1D).

### Wounding Increased Radiation-Induced γ-H2AX formation in Lin^+^ bone marrow cells

**Figure 2:**
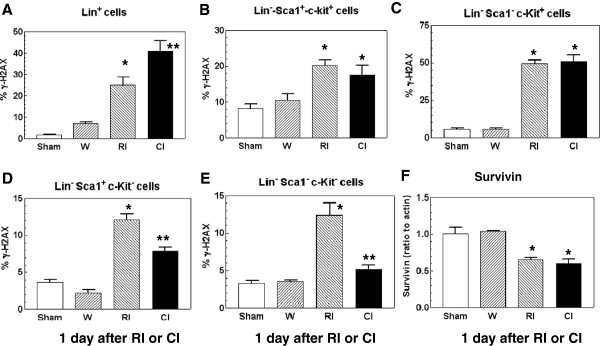
** Combined injury modulated γ-H2AX formation more than radiation injury and suppressed survivin 1 d post-irradiation and/or wounding in bone-marrow cells.** N = 8 mice per group. For panels A, D, and E: *P < 0.05 vs. sham, wounded, and CI groups; **P < 0.05 vs. sham, wounded, and RI groups. For panels B and C: *P < 0.05 vs. sham and wounded groups (W: wounded; RI: radiation injury at 8.5 Gy; CI: combined injury). For panel F: *P < 0.05 vs. sham and wounded groups (RI: radiation injury at 9.75 Gy; CI: combined injury).

Phosphorylation of histone protein H2AX on serine 139 (γ-H2AX) occurs at sites flanking DNA double-strand breaks [24]. Ataxia telangiectasia mutated (ATM) protein kinase autophosphorylates and phosphorylates histone H2AX (γ-H2AX) in response to DNA double-strand breaks [25]. Radiation increases γ-H2AX formation [26], which is a reflection of DNA double-strand breaks [24]. To determine if wounding altered the radiation-induced γ-H2AX increase, femoral bone marrow cells were harvested from mice 1 d after sham-treatment, wounding, radiation injury, and combined injury and separated into lineage-positive (Lin^+^) cells, and the lineage-negative (Lin^−^) cells, including Lin^−^-Sca1^+^-c-Kit^+^ cells, Lin^−^-Sca1^−^-c-Kit^+^ cells, Lin^−^-Sca1^+^-c-Kit^−^, and Lin^−^-Sca1^−^-c-Kit^−^. Because radiation at 9.75 Gy killed almost all of bone marrow cells, in order to collect sufficient bone marrow cells for γ-H2AX measurements, mice received 8.5 Gy, a LD_0/30_ dose to RI mice and a LD_15/30_ dose to CI mice. As shown in Figure 2, radiation significantly increased γ-H2AX formation in all five types of cells, whereas wounding alone did not. Nevertheless, wounding further increased radiation-induced γ-H2AX formation only in Lin^+^ cells by 64% (Figure 2A) but not in Lin^−^-Sca1^+^-c-kit^+^ cells (Figure 2B) and Lin^−^-Sca1^−^-c-Kit^+^ cells (Figure 2C). In contrast, wounding reduced the radiation-induced increases in γ-H2AX formation in Lin^−^-Sca1^+^-c-Kit^−^ cells by 36% (Figure 2D) and Lin^−^-Sca1^−^-c-Kit^−^ cells by 58% (Figure 2E), indicating that wound trauma enhanced H2AX phosphorylation in Lin^+^ cells but diminished H2AX phosphorylation in Lin^−^ c-kit^−^ cells.

Survivin is a protein that regulated by p53 [27] and inhibits Bax and Fas [28,29]. To determine whether ionizing radiation alone or combined with wound trauma could alter survivin, survivin from bone marrow cells collected from mice exposed to 9.75 Gy was measured using western blotting. Figure 2F shows that ionizing radiation alone suppressed survivin and wound trauma did not further its suppression, suggesting that survivin but not γ-H2AX would be a dependable biomarker for radiation dose assessment under conditions of either radiation alone or radiation combined with wound trauma.

### Wounding decreased radiation-Induced γ-H2AX formation in PBMCs

**Figure 3:**
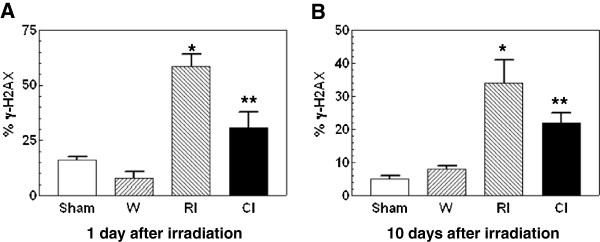
** Combined injury attenuated radiation-induced increases in γ-H2AX formation in peripheral blood mononuclear cells.** N = 8 mice per group; *P < 0.05 vs. sham, wounded, and CI groups; **P < 0.05 vs. sham, wounded, and RI groups. W: wounded; RI: radiation injury at 8.5 Gy; CI: combined injury.

Like Lin^+^ bone marrow cells, radiation significantly increased γ-H2AX formation as shown in Figure 3. Unlike Lin^+^ bone marrow cells, wounding diminished this increase by 48% 1 d (Figure 3A) and by 35% 10 d (Figure 3B) but not after 30 d (data not shown). Wounding alone did not alter the baseline of γ-H2AX formation.

### Wounding further reduced radiation-induced splenocyte decreases

**Figure 4:**
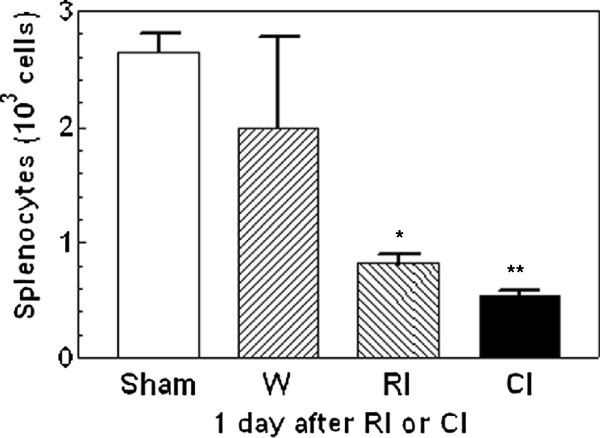
** Combined injury reduced numbers of splenocytes more than radiation injury 1 d post-irradiation and/or wounding.** N = 4 mice per group. *P < 0.05 vs. sham, wounded, and CI groups; **P < 0.05 vs. sham, wounded, and RI groups. W; wounded; RI: radiation injury at 9.75 Gy; CI: combined injury.

The spleen assists in immune function by degrading old red blood cells, storing healthy blood and assisting in detoxifying the body. As shown in Figure 4, radiation at 9.75 Gy significantly decreased the splenocyte population 1 d post-irradiation, whereas combined injury further reduced splenocyte numbers by 34% at 1 d. This difference in cell loss between radiation injured mice and combined injured mice was not observed at 7 d (data not shown).

### Wounding further reduced radiation-induced white blood cell decrease

**Figure 5:**
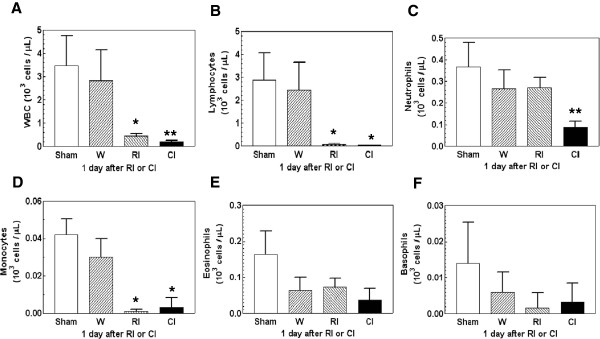
** Combined injury reduced numbers of white blood cells more than radiation injury 1 d post-irradiation and/or wounding.** N = 6 mice per group. For panels A and C, *P < 0.05 vs. sham, wounded, and CI groups; **P < 0.05 vs. sham, wounded, and RI groups. For panel B and D, *P < 0.05 vs. sham and wounded groups. Panels E and F: not significant. W; wounded; RI: radiation injury at 9.75 Gy; CI: combined injury.

It is well known that radiation reduces the circulating white blood cell (WBC) population. In this study, radiation injury at 9.75 Gy resulted in a significant reduction of total WBCs, lymphocytes, neutrophils, and monocytes 1d post-irradiation (Figure 5A-D). Numbers of total WBCs and neutrophils, but not lymphocytes and monocytes, were further reduced after combined injury by 57% and 68%, respectively (Figure 5A and C). However, eosinophils (Figure 5E) and basophils (Figure 5F) were not significantly altered by either wounding, radiation injury, or combined injury. The combined-injury-induced further cell reduction at 1d was not observed at 7d (data not shown).

### Wounding further reduced radiation-induced platelet decrease

**Figure 6:**
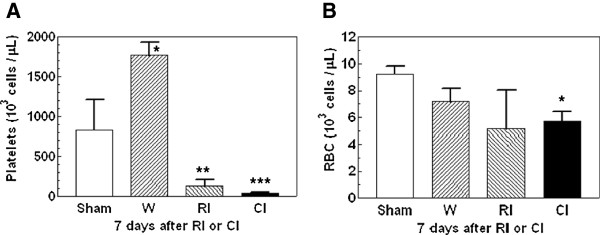
**Combined injury reduced numbers of platelets more than radiation injury 7 d post-irradiation and/or wounding.** N = 6 mice per group. For panel A, *P < 0.05 vs. sham, RI, and CI groups; **P < 0.05 vs. sham, wounded, and CI groups; ***P < 0.05 vs. sham, wounded, and RI groups. For panel B, *P < 0.05 vs. sham group. W: wounded; RI: radiation injury at 9.75 Gy; CI: combined injury.

Bone marrow is a radio-sensitive tissue. Platelet production is derived from megakaryocytes in bone marrow. In a previous study we found that the number of platelets decreased to a nadir of 30,000/μl between 8 and 10 days after 6.5 Gy ^60^Co-gamma photons [30]. In this present study, the numbers of platelets in blood of wounded, radiation injured, and combined injured mice at 1 d were not significantly different from those in sham-mice (data not shown). The number of platelets in blood was increased significantly 7d after wounding to 1765 ± 167 x 10^3^ cells/μl. In contrast, platelet numbers decreased after irradiation alone to 125 ± 86 x 10^3^ cells/μl. However, platelet numbers decreased even further after combined injury to 41 ± 15 x 10^3^ cells/μl (Figure 6A).

Unlike platelets, Figure 6 shows that 1 d (data not shown) and 7 d post-irradiation and/or wounding, red blood cells (RBCs) in peripheral blood were not altered by wounding and radiation injury, but the number of RBCs at 7 d but not 1 d (data not shown) in combined injured-mice was significantly lower than in the sham group, but not in wound and radiation injured groups (Figure 6A).

### Wounding enhances radiation-induced cytokine concentrations

**Figure 7:**
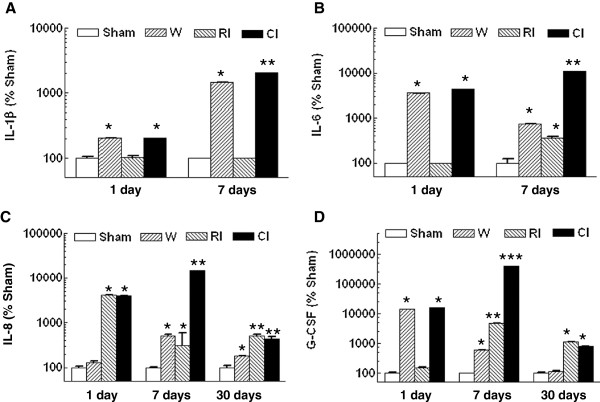
** Combined injury increased radiation-induced cytokine elevation.** N = 6 mice per group. For panel A, at 1 day: *P < 0.05 vs. sham and RI groups; at 7 days: *P < 0.05 vs. sham, RI, and CI groups; **P < 0.05 vs. sham, wounded, and RI groups. For panel B, at 1 day: *P < 0.05 vs. sham and RI groups; at 7 days: *P < 0.05 vs. sham and CI groups; **P < 0.05 vs. sham, wounded, and RI groups. For panel C, at 1 day: *P < 0.05 vs. sham and wounded groups; at 7 days: *P < 0.05 vs. sham and CI groups; **P < 0.05 vs. sham, wounded, and RI groups; at 30 days: *P < 0.05 vs. sham, RI, and CI groups; **P < 0.05 vs. sham and wounded. For panel D, at 1 day: *P < 0.05 vs. sham and RI groups; at 7 days: *P < 0.05 vs. sham, RI, and CI groups; **P < 0.05 vs. sham, wounded, and CI groups; ***P < 0.05 vs. sham, wounded, and RI groups; at 30 days: *P < 0.05 vs. sham and wounded. W; wounded; RI: radiation injury at 9.75 Gy for 1 day and 7 days and 9.25 Gy for 30 days; CI: combined injury.

Because radiation causes a short-term inflammatory response by increasing concentrations of proinflammatory cytokines in plasma [31-33] and spleen [34,35], cytokines have been considered as early biomarkers for radiation injury [21]. As shown in Figure 7, combined injury but not radiation injury induced increases in IL-1β by 98%, IL-6 by 44-fold, and G-CSF by 123-fold 1 d post-irradiation and enhanced radiation-induced increases further by 20-fold, 31-fold, and 84-fold, respectively, 7 d post-radiation, whereas IL-8 was increased by both RI and CI to the same level by 41-fold 1d after irradiation, and by only CI by 48-fold 7 d after irradiation. Thirty days later, only IL-8 and G-CSF remained above the baseline in combined injured mice, but the increases were not different from those induced by radiation alone.

## Discussion

Internationally general guidelines for early medical diagnosis and biodosimetric assessment of individuals exposed to radiation are well established [36]. These approaches usually apply for assessment of whole-body irradiation alone. However, frequently, individuals involved in radiation accidents and cancer radiation-therapy patients may experience a secondary or tertiary tissue injury. Research efforts for biodosimetry need to be developed and validated for assessment under combined injury with other confounding factors. Since time-course studies exhibited changes in blood cell numbers, cytokine concentrations in blood, protein phosphorylation in bone marrow cells and PBMCs, and clinical signs at various time points after combined injury, we assigned these changes for combined injury into three general periods: early, intermediate, and delayed expression. Most important, changes in molecular and genetic biomarkers in blood and bone marrow were significant after combined injury.

In bone marrow cells, combined injury increased γ-H2AX formation more than radiation alone 1d post-irradiation and/or wounding in Lin^+^ bone-marrow cells. Wounding alone had no impact on this biomarker, suggesting that wounding does not induce double-strand breaks in DNA and that the biomarker, γ-H2AX, is specific to radiation and combined injury (Figure 2). It is not clear why wound trauma enhanced radiation-induced γ-H2AX formation in these cells. It warrants further studies.

The enhancement occurred only in Lin^+^ bone-marrow cells but not Lin^−^ bone-marrow cells. Instead, wounding attenuated the radiation-induced γ-H2AX formation in Lin^−^-Sca1^+^-c-kit^−^ and Lin^−^-Sca1^−^-c-kit^−^ cells. It is not clear why presence or absence of c-kit receptors on bone marrow cells varied in response to combined injury. Like Lin^−^-c-kit^−^ bone-marrow cells, wounding attenuated the radiation-induced γ-H2AX formation 1 d and 10 d in PBMCs (Figure 3). Interestingly, both RI and CI significantly suppressed survivin, a protein regulated by p53 [27] and inhibiting Bax, Fas, and caspases [28,29], to a similar level. This observation made survivin potentially become a dependable biodosimeter under radiation combined with wound trauma for radiation dose assessment.

Early changes in γ-H2AX formation were also accompanied with reductions in numbers of splenocytes, lymphocytes, and neutrophils (Figures 4,5). Although bone marrow cells were altered in response to wounding, the changes in these cells did not coincide with the increase in Fms-related tyrosine kinase 3 (Flt-3) ligand concentrations in irradiated and combined injured mice. Flt-3 ligand is a hematopoietic four-helical-bundle cytokine. It is structurally homologous to stem cell factor (SCF) and colony stimulating factor 1 (CSF-1). It is a member of a small family of growth factors that stimulate the proliferation of hematopoietic cells. CD135 is its receptor and stimulates B- and T-cell proliferation and maturation [37]. In synergy with other growth factors, Flt-3 ligand stimulates the proliferation and differentiation of various blood cell progenitors [38,39]. Flt-3 ligand plasma concentrations are inversely correlated with either the frequency of colony-forming cells in the bone marrow or the extent of bone marrow aplasia postirradiation [38,40,41]. This laboratory previously reported that radiation increased Flt-3 ligand concentrations 3-fold 1 d post-irradiation, whereas subsequent wounding did not further increase it [42], indicating that wounding is not associated with bone marrow aplasia. The result suggested that Flt-3 ligand is a dependable biodosimeter under radiation combined with wound trauma as well for radiation dose assessment.

Intermediate biomarkers for combined injury are reductions of the numbers of RBCs and platelets (Figure 6). The latter was induced by radiation injury to bone marrow as shown previously [30]. Wounding used remaining platelets in the clotting process to reduce bleeding thereby decrease the number even further after irradiation. The reduction occurred 7 d (but not 1 d) after combined injury, whereas wounding alone induced an increase in numbers of platelets. The difference in early biomarkers such as numbers of WBCs, neutrophils, and splenocytes was no longer measurable in combined injured mice after Day 7. The degree of reduction of these cells was similar between radiation alone and combined injury.

Additionally, combined injured mice displayed magnified increases in IL-1β, IL-6, IL-8, and G-CSF in serum on day 1, whereas radiation-induced cytokine changes in serum occurred only in IL-8 (Figure 7). The synergistic increases in IL-1β, IL-6, IL-8, and G-CSF concentrations in serum were greater on 3–7 d [4] than those increases observed on 1 d. Combined injury had a greater effect on concentrations of these cytokines than did radiation alone. A similar synergy in IL-6 concentrations was observed in combined radiation and burn mouse model [43].

The increased concentrations of IL-1β, IL-6, IL-8, and G-CSF 7 days after combined injury were caused by the combined injury-induced sepsis due to the bacterial entry from the broken GI barrier and the skin-wound site. The sepsis began 3 days after combined injury [4]. Therefore, these magnified increases were not observed 1 day but became statistically significant 7 days after combined injury compared to wound trauma alone [4].

These increases in serum cytokines are a natural response to injury in an attempt to control damage to tissue and restore homeostasis. IL-1β and IL-6 are proinflammatory but the usual responding cells have been killed by radiation. G-CSF promotes hemopoietic differentiation of progenitor cells into granulocytic cells, including neutrophils, but the progenitor cells have also been killed. IL-8 promotes chemotaxis of neutrophils to a site of injury but there are no neutrophils to respond. So, these increases in serum cytokines are ineffective while they indicate a response to injury early after injury. Therefore, increasing values during early hours and days following combined injury may provide a strong indication for intensive therapy. Thirty days after irradiation or combined injury, IL-8 and G-CSF concentrations in the serum remained above the baseline. Perhaps continuously increasing high concentrations of either IL-8 or G-CSF in the blood indicate individuals that are likely to survive but other biomarkers are needed for definitive early prognosis.

Skin-wound closure (as an indicator of healing) is known to depend on the dose of radiation [44,45]. Thus, wound closure is an indicator for the extent of tissue injury and healing rate in combined injured mice. In non-irradiated mice, skin-wounds healed completely within 15 days. In combined injured mice given a dose of 9.75 Gy, more than 30 days were needed for complete closure in survivors, which indicated a delayed healing rate. (Figure 1D).

The nadirs of body weight loss may also be a useful parameter for distinguishing radiation injury from combined injury. In our study, the nadirs for the body weight in radiation-injured mice and combined-injured mice were 22 days and 14 days, respectively (Figure 1C).

A combination of biomarkers and clinical signs and symptoms could be useful for determining and differentiating the contribution of dose of radiation to survival with or without tissue injury. This is a continuing working hypothesis. Blood components and serum cytokine concentrations are valuable biomarkers of tissue injury and should be closely monitored for establishing radiation dose assessment combined with wounding. Validation of selected blood biomarkers for prognosis of radiation injury and dose assessment await further rigorous specificity and sensitivity studies. These studies should use appropriate animals, when possible, and address potential relevant confounding effects, for instance, age, gender, chronic disease, burn, inflammation, wounding, and partial-body exposure.

This current study (Figure 1A) corroborates that radiation combined with wound trauma potentiates lethality compared to radiation alone [5]. Studies with several animal species have shown similar results [6-10,33,34]. In addition to lethality, a synergistic effect on behavior or emesis after radiation combined with daily exhaustive exercise, continuous exposure to cold (6°C), or continuous exposure to high altitude (15,000 feet) are documented [46]. Furthermore, Kato and Schull [47] reported an increased mortality rate from lung cancer related to cigarette smoking in atomic-bomb survivors. It would be of interest to investigate whether a similar finding between acute and long-term effects after radiation injury combined with wound trauma is present.

Failure to thrive early after injury indicates a need to initiate aggressive early therapy. Combined injured mice drank more water than sham-treated mice. In contrast, radiation alone suppressed water consumption. Hypodipsia was observed 1 day after irradiation and lasted for 6 days (Figure 1B). Then, the volume of daily water intake in both irradiated and combined injured mice returned to the normal baseline as found in sham-treated mice. Therefore, daily water consumption volume distinguishes radiation injury alone from combined injury and, so, might be a good indicator for prognosis. The return to normal consumption of food and water that is reflected in increased body weight may be a good prognostic indicator of recovery from radiation injury and combined injury. Table 1 summarizes the signs.

**Table 1:** Combined injury signs compared to radiation injury signs

Early signs	More water intake
	Greater decreases in numbers of total WBCs
	More γ-H2AX formation in Lin^+^ bone-marrow cells
	Similar decreases in survivin protein
	Increased serum concentrations of IL-1β, -6, -8, G-CSF, and Flt-3 ligand
Intermediate signs	Faster body-weight decreases
	Slower wound-healing rate
	Greater decreases in numbers of RBCs and platelets
	Less γ-H2AX formation in PBMCs
	Greater increases in serum concentrations of IL-1β, -6, -8, and G-CSF
Late signs	Sustained elevated serum concentrations of IL-8 and G-CSF
	More mortality
	Slower wound-healing rate

In summary, we demonstrated for the first time that responses of selected biomarkers to radiation were changed significantly by subsequent wounding shortly after irradiation. The early biomarkers (1 d after injury) included enhanced responses in γ-H2AX formation in Lin^+^ bone marrow cells, numbers of splenocytes, lymphocytes, and neutrophils, and concentrations of IL-1β, IL-6, IL-8, and G-CSF in blood. Increased water consumption by mice also occurred early after combined injury. The intermediate biomarkers (7 – 10 d after injury) included continued large increases in IL-1β, IL-6, IL-8, and G-CSF concentrations in blood and reduced γ-H2AX formation in PBMCs. Large decreases in body weight and decreased wound healing were notable during the intermediate period. Although continued elevation of IL-8 and G-CSF concentrations and slow wound healing persisted at 30d, other biomarkers in the same mice had recovered to normal values in the surviving mice.

## Conclusion

The severity of injury manifested immediately after irradiation combined with wound injury, and prognosis for its survival depends upon a complex matrix of factors including numbers of circulating blood cells, molecular biomarkers, and other physiological endpoints. Changes in the parameters measured in this study support not only early intervention with intensive therapy to strengthen chances of survival but also the concept of successful therapy, which may be adjusted to conserve critical medical resources. Biomarkers such as levels of survivin in bone marrow cells and Flt-3 ligand in serum are dependable ones for radiation dose assessment under either radiation alone condition or radiation combined with wound trauma condition. Furthermore, the greater amount of water consumption, the high speed of wound healing rate, the low levels of serum cytokine concentrations can distinguish the wounded subjects from those also exposed to irradiation. Therefore, the critical medical resources can be dedicated as needed.

## Methods

### Animals

B6D2F1/J female mice were purchased from Jackson Laboratory (Bar Harbor, ME) and used when 12–20 wk old. Male mice were not used in this study because of aggression, which in these experiments could lead to further damage to wound sites and enhanced infection. All mice were randomly assigned to experimental groups. Eight mice were housed per filter-topped polycarbonate cage (MicroIsolator) in conventional holding rooms. Rooms were provided 20 changes of 100% fresh air per h, conditioned to 72 ± 2°F with relative humidity of 50 ± 20%. Mice were maintained on a 12-h 6 am light/6 pm dark, full-spectrum-light cycle with no twilight. Research was conducted in a facility accredited by the Association for Assessment and Accreditation of Laboratory Animal Care-International (AAALAC-I). All procedures involving animals were reviewed and approved by the AFRRI Institutional Animal Care and Use Committee. Euthanasia was carried out in accordance with recommendations [48,49] and guidelines [50].

Prior to experiments, hair of the dorsal surface of mice was removed under anesthesia (methoxyflurane inhalation) using electric clippers. Mice were placed in well-ventilated acrylic restrainers for irradiation or sham treatments. Within 1 h after irradiation or sham irradiation, mice were anesthetized by methoxyflurane inhalation, and wounding or sham wounding was performed. All mice received an i.p. injection of 0.5 ml sterile isotonic 0.9% NaCl as fluid therapy immediately after sham handling, irradiation, and/or wounding. After fluid therapy, mice were returned to their original cages. After irradiation and/or wounding, their water consumption for the first 7 days, body weights, wound closure, and survival for 30 days were measured as described previously [4].

### Irradiation

Mice were given selected doses of whole-body ^60^Co γ-photon radiation delivered at a dose rate of 0.4 Gy/min bilaterally. Dosimetry was performed using the alanine/electron paramagnetic resonance system. Calibration of the dose rate with alanine was directly traceable to the National Institute of Standards and Technology and the National Physics Laboratory of the United Kingdom. Mice treated as shams were treated identically to other groups but received no radiation and were not wounded.

### Skin wounding

Within 1 h after irradiation, mice were anesthetized by methoxyflurane prior to wounding. A non-lethal total body surface area (TBSA) wound was administered approximately 20 mm from the occipital bone and between the scapulae using a stainless steel punch on a Teflon-covered board cleaned with 70% alcohol before each use. The panniculus carnosus muscle and overlying skin (approximately 24 mm in length and about 15 mm in width) were removed. Mice receiving only sham treatments were manipulated identically to other groups but received no radiation or wounding. Mice receiving only wounds were also treated identically to other groups but received no radiation.

### Collection of bone marrow and blood

In a separate experiment, mice were anesthetized with methoxyflurane 1, 10, and 30 d post-radiation and/or wounding. Blood was collected through cardiac puncture. Mice were euthanized and bone marrow cells from femurs were collected. Samples at the 30-d time point were collected from mice at the end of the survival experiment.

### Peripheral blood mononuclear cell staining

Blood samples were collected 1 d and 10 d after RI and CI. According to the manufacturer's protocol provided by Invitrogen, whole blood samples were incubated with ACK lysing buffer (Invitrogen) at 37°C for 15 min to remove red blood cells. Then by following the manufecturer’s protocol provided by BD Biosciences, obtained peripheral blood mononuclear cells (PBMCs) were washed with PBS, incubated with 1 μl of Live/Dead Fixable Dead Cell Stain-Near-Infrared (Invitrogen) and <1 μg/ml of Mouse Fc Block (BD Biosciences) in PBS on ice for 30 min, washed with PBS, incubated on ice for 30 min with anti-mouse-CD45-allophycocyanin (e-Bioscience), permeabilized and stained with perm/wash buffer (BD Biosciences), containing 7% v/v anti-phospho-H2AX-FITC (Millipore) and Mouse Fc block (BD Biosciences) on ice for 20 min, and washed with 1x perm/wash buffer (BD Biosciences).

### Bone marrow cell staining

According to the manufacturer’s protocol provided by BD Biosciences, approximately 1 × 10^6^ total bone marrow cells were incubated with 1 μl of Live/Dead Fixable Dead Cell Stain-Near-Infrared (Invitrogen) and <1 μg/ml of Mouse Fc Block (BD Biosciences) in PBS on ice for 30 min and washed with PBS. The cells were then incubated with a cocktail of Mouse Hematopoietic Lineage Flow Cocktail-eFluor450 (e-Bioscience), anti-mouse-c-kit-Cy5(e-Bioscience), and anti-mouse-Sca-1-phycoerythrin-Cy5.5(e-Bioscience), and then washed again with PBS, fixed with 1.5% paraformaldehyde for 20 min on ice, washed with PBS, permeabilized and stained with perm/wash buffer (BD Biosciences), containing 7% v/v anti-phospho-H2AX-FITC (Millipore) and Mouse Fc block (BD Biosciences), and washed with 1x perm/wash buffer (BD Biosciences).

### Flow cytometry for γ-H2AX measurement

Stained blood and bone marrow cells with no less than 5,000 cells were analyzed on a LSR II cytometer (BD Biosciences) equipped with 407, 488 and 633 nm lasers, emission filters for near-infrared, FITC, PE-Cy5.5, APC, and Cy5, and a high-throughput sampler in Biomedical Instrumentation Center (BIC) in USUHS. Data were analyzed with FACSDiva software (BD Biosciences). Analyses were performed at the Uniformed Services University of the Health Sciences Flow Cytometry Core Facility, Bethesda, Maryland.

### Assessment of blood cell profile in peripheral blood

The ADVIA 2120 Hematology System (Siemens, Deerfield, IL) was employed for assessment of blood samples collected 1 d and 7 d after RI and CI. Differential analysis was conducted using the peroxidase method and the light scattering techniques recommended by the manufacturer.

### Splenocyte measurements

Spleens were collected from each mouse 1 d and 7 d after RI and CI. Each specimen was mashed in a cell strainer (BD Falcon, Bedford, MA) with 1x HBSS. Splenocytes in the buffer were washed with 1x ACK lysis buffer (Invitrogen), vortexed with a shaker, and centrifuged at 800 xg. Splenocytes were collected and counted using a hemocytometer.

### Serum cytokine measurements

Cytokines in serum were measured in mice 1, 7 d, and 30 d after sham treatment, wounding, RI, or CI (n = 6 mice per group at each time point). Whole blood (0.7 – 1 ml) was collected by cardiac puncture from mice anesthetized with methoxyflurane. Serum was separated by centrifugation at 1000xg and stored at −70°C until assayed. Cytokine protein concentrations in serum were analyzed using the Bio-Plex ^TM^ Cytokine Assay (Bio-Rad; Hercules, CA) to analyze a panel of 23 cytokines (Mouse Cytokine 23-Plex; Bio-Rad) following the manufacturer’s directions. Briefly, sera from each animal (6 animals per group) were diluted fourfold and examined in duplicate. Data were analyzed using the Luminex® 100™ System (Luminex Corp., Austin, TX), quantitated using MiraiBio MasterPlex® CT and QT Software (Hitachi Software Engineering America Ltd.; San Francisco, CA), and concentrations expressed in pg/ml. The cytokines analyzed were: IL-1α, IL-1β, IL-2, IL-3, IL-4, IL-5, IL-6, IL-8 (i.e. KC), IL-9, IL-10, IL-12(p40), IL-12(p70), IL-13, IL-17, Eotaxin, G-CSF, GM-CSF, IFN-γ, MCP-1, MIP-1α, MIP-1β, RANTES, and TNF-α.

### Endotoxin measurements

All materials including water, phosphate saline buffer, and saline, which were used for evaluation of test samples collected from mice, were tested with Endosafe^®^ (Charles River Endosafe, Charleston, SC) to ensure that they were endotoxin-free according to the manufacturer’s procedure.

### Statistical analysis

Data are expressed as the mean ± s.e.m. For each experiment, 6 mice per group were tested on an individual basis. Each biochemical assay was performed in duplicate while the blood cell differentiation test was performed once. One-way ANOVA, two-way ANOVA, studentized-range test, Bonferroni’s inequality, and Chi-square test were used for comparison of groups, with 5% as a significant level.

## Abbreviations

WBCs: White blood cells; RBCs: Red blood cells; IL: Interleukin; Flt-3, Fms-related tyrosine kinase 3; W: Wound; RI: Radiation injury; CI: Combined injury; TBSA: Total body surface area; VSD: Veterinary science department.

## Competing interest

The authors declare that they have no competing interests.

## Authors’ contributions

JGK, TBE, and GDL conceived and designed the experiments and wrote the manuscript. BRG, TMB, MZ, ICD, PHN, LHC, and RF performed all the experiments in the manuscript. All authors read and approved the final manuscript.

## References

[B1] Kishi HS Effects of the “special bomb”: recollection of a neurosurgeon in Hiroshima, August 8–15, 1945 Neurosurgery 2000 47 441 6 10.1097/00006123-200008000-00034 10942018

[B2] Iijima S Pathology of atomic bomb casualties Acta Pathol Jpn 1982 32 Suppl. 2 237 70 7187578

[B3] Barabanova AV Significance of beta-radiation skin burns in Chernobyl patients for the theory and practice of radiopathology Vojnosanit Pregl 2006 63 477 80 10.2298/VSP0605477B 16758799

[B4] Kiang JG Jiao W Cary L Mog SR Elliott TB Pellmar TC Ledney GD Wound trauma increases radiation-induced mortality by increasing iNOS, cytokine concentrations, and bacterial infections Radiat Res 2010 173 319 32 10.1667/RR1892.1 20199217 PMC10113926

[B5] Ledney GD Elliott TB Combined injury: Factors with potential to impact radiation dose assessments Health Physics 2010 98 145 52 10.1097/01.HP.0000348466.09978.77 20065676

[B6] Davis AK Alpen EL Sheline GE The combined effects of thermal burns and whole-body x-radiation on survival time and mortality Ann Surg 1954 140 113 8 10.1097/00000658-195407000-00013 13159151 PMC1609615

[B7] Valeriote FA Baker DG The combined effects of thermal trauma and x-irradiation on early mortality Radiat Res 1964 22 693 702 10.2307/3571550 14201877

[B8] Korlof B Infection of burns, I. A bacteriological and clinical study of 99 cases. II. Animal experiments: burns and total body x-irradiation Acta Chir Scand Suppl 1956 209 1 144 13326153

[B9] Brooks JW Evans EI Ham WT Reid JD The influence of external body radiation on mortality from thermal burns Ann Surg 1952 136 533 45 14953182 10.1097/00000658-195209000-00018 PMC1802884

[B10] Baxter H Drummond JA Stephens-Newsham LG Randall RG Studies on acute total body irradiation in animals. I. Effect of streptomycin following exposure to a thermal burn and irradiation Plast Reconstr Surg 1953 12 439 45 10.1097/00006534-195312000-00007 13111923

[B11] McDonnel GM Crosby WH Tessmer CF Moncrief WH Baker HJ Goldstein JD Woodward K Shively JN Daniell HW Horava A Claypool HA Effects of nuclear detonations on a large biological specimen (swine) Report WT-1428, Operation Plumbbob, Project 4.1, Defense Atomic Support Agency, Sandia Base, Albuquerque, New Mexico 1961

[B12] Ledney GD Elliott TB Moore MM Modulations of mortality by tissue trauma and sepsis in mice after radiation injury 1992 Baltimore: K.L. Mossman and W.A. Mills, Eds, Williams and Wilkins 202 217

[B13] Zou Z Sun H Su Y Cheng T Luo C Progress in research on radiation combined injury in China Radiat Res 2008 169 722 9 10.1667/RR1284.1 18494547

[B14] Koenig KL Goans RE Hatchett RJ Mettler FA Schumacher TA Noji EK Jarrett DG Medical treatment of radiological casualties: current concepts Ann Emerg Med 2005 45 643 52 10.1016/j.annemergmed.2005.01.020 15940101

[B15] Lausevic Z Lausevic M Trbojevic-Stankovic J Krstic S Stojimirovic B Predicting multiple organ failure in patients with severe trauma Can J Surg 2008 51 97 102 18377749 PMC2386337

[B16] Houtgraaf JH Versmissen J van der Giessen WJ A concise review of DNA damage checkpoints and repair in mammalian cells Cardiovasc Revasc Med 2006 7 165 72 10.1016/j.carrev.2006.02.002 16945824

[B17] Kiang JG Garrison BR Gorbunov NV Radiation combined injury: DNA damage, apoptosis, and autophagy Adapt Med 2010 2 1 10 10.4247/AM.2010.ABA004 PMC8491956 34616567

[B18] Redon CE Nakamura AJ Gouliaeva K Rahman A Blakely WF Bonner WM The use of gamma-H2AX as a biodosimeter for total-body radiation exposure in non-human primates PLoS One 2010 5 e15544 10.1371/journal.pone.0015544 21124906 PMC2990755

[B19] Walden TL Farzaneh NK Biological assessment of radiation damage 1989 USA: eds. Zajtchuk R., Jenkins DP, Bellamy RF, Ingran VM), TMM publications, Office of the Surgeon General, Department of the Army 85 103

[B20] Ossetrova NI Sandgren DJ Gallego S Blakely WF Combined approach of hematological biomarkers and plasma protein SAA for improvement of radiation dose assessment triage in biodosimetry applications Health Phys 2010 98 204 8 10.1097/HP.0b013e3181abaabf 20065684

[B21] Partridge MA Chai Y Zhou H Hei TK High-throughput antibody-based assays to identify and quantify radiation-responsive protein biomarkers Int J Radiat Biol 2010 86 321 8 10.3109/09553000903564034 20353341 PMC3664638

[B22] Ossetrova NI Blakely WF Multiple blood-proteins approach for early-response exposure assessment using an in vivo murine radiation model Int J Radiat Biol 2009 85 837 50 19863200

[B23] Messerschmidt O Kombinationsschaden als folge nuklearer explosionen 1977 Munchen: R. Zenker, F Deucher, and W. Schink, Eds, Urban and Schwarzenberg 1 54

[B24] Rogakou EP Pilch DR Orr AH Ivanova VS Bonner WM DNA double-stranded breaks induce histone H2AX phosphorylation on serine 139 J Biol Chem 1998 273 5858 68 10.1074/jbc.273.10.5858 9488723

[B25] Burma S Chen BP Murphy M Kurimasa A Chen DJ ATM phosphorylates histone H2AX in response to DNA double-strand breaks J Biol Chem 2002 276 42462 7 11571274 10.1074/jbc.C100466200

[B26] Fukumoto R Kiang JG Geldanamycin analog 17-DMAG limits apoptosis in human peripheral blood cells by inhibition of p53 activation and its interaction with heat-shock protein 90 kDa after exposure to ionizing radiation Radiat Res 2011 176 333 45 10.1667/RR2534.1 21663398 PMC4076157

[B27] Mirza A McGuirk M Hockenberry TN Wu Q Ashar H Black S Wen SF Wang L Kirschmeier P Bishop WR Nielsen LL Pickett CB Liu S Human survivin is negatively regulated by wild-type p53 and participates in p53-dependent apoptotic pathway Oncogene 2002 21 2613 22 10.1038/sj.onc.1205353 11965534

[B28] Tamm I Wang Y Sausville E Scudiero DA Vigna N Oltersdorf T Reed JC IAP-family protein survivin inhibits caspase activity and apoptosis induced by Fas (CD95), Bax, caspases, and anticancer drugs Cancer Res 1998 58 5315 20 9850056

[B29] Shin S Sung BJ Cho YS Kim HJ Ha NC Hwang JI Chung CW Jung YK Oh BH An anti-apoptotic protein human survivin is a direct inhibitor of caspase-3 and −7 Biochemistry 2001 40 1117 23 10.1021/bi001603q 11170436

[B30] Elliott TB Brook I Stiefel SM Quantitative Study of Wound Infection in Irradiated Mice Int J Radiat Biol 1990 58 341 50 10.1080/09553009014551671 1974580

[B31] Wickremesekera JK Chen W Cannan RJ Stubbs RS Serum proinflammatory cytokine response in patients with advanced liver tumors following selective internal radiation therapy (SIRT) with (90) Yttrium microspheres Int J Radiat Oncol Biol Phys 2001 49 1015 21 10.1016/S0360-3016(00)01420-6 11240242

[B32] Wichmann MW Meyer G Adam M Detrimental immunologic effects of preoperative chemoradiotherapy in advanced rectal cancer Dis Colon Rectum 2003 46 875 87 10.1007/s10350-004-6677-z 12847360

[B33] Barthelemy-Brichant N Bosquee L Cataldo D Increased IL-6 and TGF-beta 1 concentrations in bronchoalveolar lavage fluid associated with thoracic radiotherapy Int J Radiat Oncol Biol Phys 2004 58 758 67 10.1016/S0360-3016(03)01614-6 14967431

[B34] Peterson VM Adamovicz JJ Elliott TB Moore MM Madonna GS Jackson WE Ledney GD Gause WC Gene expression of hematoregulatory cytokines is elevated endogenously after sublethal gamma irradiation and is differentially enhanced by therapeutic administration of biologic response modifiers J Immunol 1994 153 2321 30 8051428

[B35] Singh VK Grace MB Jacobsen KO Chang CM Parekh VI Inal CE Shafran RL Whitnall AD Kao TC Jackson WE Whitnall MH Administration of 5-androstenediol to mice: Pharmacokinetics and cytokine gene expression Exp Mol Pathol 2008 84 178 88 10.1016/j.yexmp.2007.12.001 18262521

[B36] Alexander GA Swartz HM Amundson SA Blakely WF Buddemeier B Gallez B Dainiak N Goans RE Hayes RB Lowry PC Noska MA Okunieff P Salner AL Schauer DA Trompier F Turteltaub KW Voisin P Wiley AL Wilkins R BiodosEPR-2006 Meeting: acute dosimetry consensus committee recommendations on biodosimetry applications in events involving uses of radiation by terrorists and radiation accidents Radiat Measurements 2007 42 972 96 10.1016/j.radmeas.2007.05.035

[B37] Lyman SD Biology of flt3 ligand and receptor Int J Hematol 1995 62 63 7 8590775 10.1016/0925-5710(95)00389-a

[B38] Prat M Demarquay C Frick J Dudoignon N Thierry D Bertho JM Use of flt3 ligand to evaluate residual hematopoiesis after heterogeneous irradiation in mice Radiat Res 2006 166 504 11 10.1667/RR0568.1 16953669

[B39] Shurin MR Esche C Lotze MT FLT3: receptor and ligand. Biology and potential clinical application Cytokine Growth Factor Rev 1998 9 37 48 10.1016/S1359-6101(97)00035-X 9720755

[B40] Huchet A Belkacemi Y Frick J Prat M Muresan-Kloos I Altan D Chapel A Gorin NC Gourmelon P Bertho JM Plasma Flt-3 ligand concentration correlated with radiation induced bone marrow damage during local fractionated radiotherapy Int J Radiat Oncol Biol Phys 2003 57 508 15 10.1016/S0360-3016(03)00584-4 12957264

[B41] Wodnar-Filipowicz A Lyman SD Gratwohl A Tichelli A Speck B Nissen C Flt3 ligand level reflects hematopoietic progenitor cell function in aplastic anemia and hemotherapy induced bone marrow aplasia Blood 1996 88 4493 9 8977241

[B42] Jiao W Kiang JG Cary LH Elliott TB Pellmar TC Ledney GD COX-2 inhibitors are contraindicated for treatment of combined injury Radiat Res 2009 172 686 697 10.1667/RR1581.1 19929415

[B43] Palmer JL Deburghgraeve CR Bird MD Hauer-Jensen M Kovacs EJ Development of a combined radiation and burn injury model J Burn Care Res 2011 32 317 23 10.1097/BCR.0b013e31820aafa9 21233728 PMC3062624

[B44] Withers HR The dose-survival relationship for irradiation of epithelial cells of mouse skin Br J Radiol 1967 40 187 94 10.1259/0007-1285-40-471-187 6019041

[B45] Van Den Aardweg GJMJ Morris GM Bywaters A Bakker EJ Mooi WJ Changes in epidermal radiosensitivity with time associated with increased colony numbers Br J Radiol 2001 74 434 44 11388992 10.1259/bjr.74.881.740434

[B46] Kimeldorf DJ Hunt EL Ionizing radiation: Neural function and behavior 1965 Academic Press: New York and London

[B47] Kato H Schull WJ Studies of the mortality of A-bomb survivors. 7. Mortality, 1950–1978: Part I. Cancer mortality Radiat Res 1982 90 395 432 10.2307/3575716 7079470

[B48] Montgomery CA Oncologic and toxicologic research: Alleviation and control of pain and distress in laboratory animals Cancer Bulletin 1990 42 230 37

[B49] Tomasivic SP Coghlan LG Gray KN Mastromarino AJ Travis EL IACUC evaluation of experiments requiring death as an end point: A cancer center's recommendations Lab Animal January/February 1988 31 4

[B50] American Veterinary Medical Association Report of the AVMA Panel on Euthanasia J Am Veterinary Med Assoc 2001 218 669 96 10.2460/javma.2001.218.669 11280396

